# Bilateral Hip Arthroplasty: When Is It Safe to Operate the Second Hip? A Systematic Review

**DOI:** 10.1155/2018/3150349

**Published:** 2018-02-28

**Authors:** Meilyn Muskus, Jorge Rojas, Camilo Gutiérrez, Juan Guio, Guillermo Bonilla, Adolfo Llinás

**Affiliations:** ^1^Orthopedic and Traumatology Department, Hospital Universitario Fundación Santa Fe de Bogotá, Bogotá, Colombia; ^2^Orthopedic and Traumatology Department, Hospital Infantil Universitario de San José, Bogotá, Colombia; ^3^School of Medicine, Universidad de Los Andes, Bogotá, Colombia; ^4^School of Medicine, Universidad del Rosario, Bogotá, Colombia

## Abstract

**Introduction:**

Patients with degenerative hip disease frequently present with bilateral involvement that requires surgical management. The main goal when treating these patients is to achieve the maximum efficiency without increasing risk of perioperative complications; therefore, the decision regarding the best moment to operate the second hip becomes relevant. Although studies have addressed this topic, whether a simultaneous or staged surgery should be performed remains controversial. The purpose of this study was to determine, based on available evidence, the optimum strategy in terms of safety to operate the second hip in patients with bilateral involvement.

**Materials and Methods:**

A meta-analysis was planned. A systematic review of the literature was performed including clinical trials or observational analytical studies comparing the safety of bilateral arthroplasty performed simultaneously or staged by measuring major and minor complications. The appropriateness of a meta-analysis was evaluated through the detailed analysis of the risk of bias and clinical heterogeneity of the included studies.

**Results:**

Thirteen studies were selected after the systematic review. A wide variability in the methodological designs was found with a critical risk of bias in most of them. Considerable heterogeneity was detected in defining staged surgery in the cointerventions and how the outcomes were defined and measured. In response to these findings, a meta-analysis was considered not appropriate. The results showed no differences in the risk of mortality or systemic complications in young and healthy patients between simultaneous or staged surgeries. However, increased risk of complications for staged surgeries performed during the same hospitalization was observed.

**Conclusions:**

Available evidence is very heterogeneous and the quality of evidence is low. The available evidence supports the performance of simultaneous hip arthroplasty in selected patients (not older than 65 years, ASA 1-2, without cardiovascular comorbidities) and suggests the avoidance of staged surgeries within the same hospitalization.

## 1. Introduction

Bilateral hip disease is a frequent finding that can occur in up to 42% of the population with osteoarthrosis [1], and it is estimated that 25% of patients with osteoarthritis requiring total hip replacement will need a bilateral replacement [2]. This has led to the evaluation of the best strategy to operate a bilateral hip replacement without increasing the risk of perioperative comorbidities, having as options a simultaneous bilateral hip replacement or a sequential surgery [3].

The implementation of simultaneous bilateral procedures has been on the rise in recent years, arguing that it is a procedure with potential advantages such as cost reduction [2, 4], shorter rehabilitation time [5, 6], and shorter hospital length of stay [5], without presenting an increased risk of complications. Some studies have reported similar rates of deep vein thrombosis [7], pulmonary embolism [8], infection [9], and mortality [10] for simultaneous surgery when compared with two-stage procedures. On the other hand, other studies have shown simultaneous surgery to be associated with an elevated risk of blood transfusion [3, 11] and some authors recommend reserving this approach for patients with a high preoperative hemoglobin level [6]. Furthermore, at least one additional report has demonstrated an increase in the rate of complications when simultaneous surgery is performed [12]. In an attempt to clarify this information, a recent meta-analysis was performed [13]; however, its strong methodological limitations limit the applicability of the reported results [14]. In summary, to our knowledge it is not clear which surgical strategy is optimal to achieve the lowest rate of complications, in order to offer the patient the best balance between safety and efficacy.

Since available evidence is affected by a strong risk of bias, it is necessary to perform a thorough analysis of this controversial information searching for the best strategy to provide patients with a prompt relief of symptoms without increasing the risk of the aforementioned complications. Therefore, the aim of the present study is to determine which patients are suitable for a simultaneous bilateral hip arthroplasty, and, for those who are not, which moment is the safest to operate the second hip.

## 2. Materials and Methods

This systematic review was performed in accordance with the guidelines for Preferred Reporting Items for Systematic Reviews and Meta-Analyses.

### 2.1. Eligibility Criteria

Randomized clinical trials or observational analytic studies (prospective cohort-type, case-control, registry) comparing the safety of bilateral total hip replacement performed simultaneously (one stage under the same anesthesia event) or staged (two stages under different anesthesia events) in patients older than 18 years and reporting major and minor complications (mortality, thromboembolic disease, cardiovascular complications, bleeding, transfusion, gastrointestinal complications, neurological complications, infection, other complications) were considered for inclusion. Only studies published in English were included. We excluded studies of patients with malignant disease and resurfacing procedures. We excluded no studies on the basis of time of follow-up or publication date.

### 2.2. Search Strategy and Study Selection Process

The following electronic databases were examined: Specialized Register of the Cochrane Bone Group, Joint and Muscle Trauma (September 2016), Cochrane Central Register of Controlled Trials (CENTRAL) (The Cochrane Library 2016), MEDLINE (from 1950 to the third week of September 2016), Embase (from 1988 to 2016 week 37). We searched the following terms: “hip” and “arthroplasty or replacement,” “one-stage or two-stage” or “simultaneous or staged.” The search strategies for MEDLINE, CENTRAL, and Embase are shown in Appendices A, B, and C. We scrutinized the bibliography of included articles, reviews, and textbooks for potentially relevant references.

Two review authors (MM and GB) independently reviewed all the citations retrieved using the strategy described above, selecting potentially eligible studies for inclusion based on the title and abstract in the first stage and based on the complete report in the second stage. In cases of disagreement, inclusion of the study was discussed by the entire group.

### 2.3. Variables and Data Collection Process

Two review authors (MM and JG) extracted data using a standardized format that was designed for this review. We obtained the following data from the studies: author and year of the study, methodological design, years during patient enrollment, type of arthroplasty and surgical approach, number of participants, age, gender, inclusion and exclusion criteria, loss to follow-up, primary and secondary outcomes (mortality, thromboembolic disease, cardiovascular complications, bleeding, transfusion, gastrointestinal complications, neurological complications, infection, other complications).

A detailed evaluation of the characteristics of the included studies with respect to the population, interventions, definition, and measurement of the outcomes was independently assessed by two authors, to establish suitability of evidence for a meta-analysis.

### 2.4. Assessment of Study Quality

Risk of bias in the included studies was independently assessed by two review authors (JR and GB). The ROBINS-I tool “The Risk Of Bias In Non-randomized Studies - of Interventions (ROBINS-I) assessment tool” was used for the nonrandomized observational studies [15]. We classified each domain and global risk of bias in accordance with the tool as low, moderate, serious, critical, or uncertain. Nine of the studies had a “critical risk of bias.” In accordance with this tool a critical risk of bias study is too problematic to provide any useful evidence and should not be included in any synthesis. One study had a “serious risk of bias” meaning that the study has some important problems and two studies had a moderate risk of bias meaning that the study provides sound evidence for a nonrandomized study but cannot be considered comparable to a well performed randomized trial (Table 1).

The tool “Risk of Bias” was used for the randomized controlled trial according to Higgins et al. 2011 [16]. Each criterion was explicitly judged in accordance with the tool as being at either low, high, or unclear risk of bias. The overall risk of bias of the only randomized controlled study was high, meaning that bias may alter the results seriously (Table 2).

## 3. Results

Results are reported in three main sections: Selected Studies, Quality of Available Evidence, and Results of Systematic Review.

### 3.1. Selected Studies

**Figure 1:**
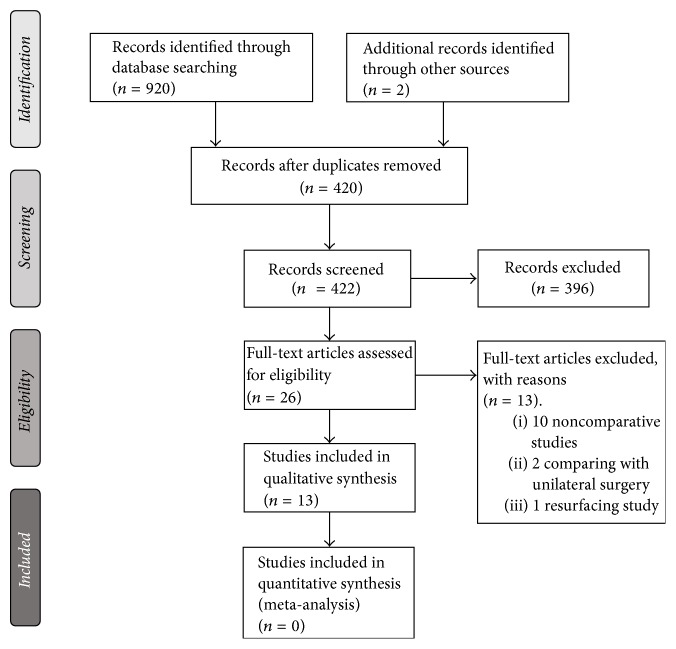
Flowchart showing selection process of available evidence.

The search strategy identified a total of 920 articles; two articles were obtained from other sources (review studies bibliography). We reviewed the full text of 26 potentially eligible studies, yielding a total of 13 studies published between 1996 and 2015 [3, 5, 7, 10, 11, 17–24] for inclusion in the systematic review. Thirteen studies were excluded, most of which due to comparisons with unilateral hip arthroplasty. A flowchart summarizing the selection process is presented in Figure 1. Among the 13 included studies, there was only one randomized clinical trial [19]. And the most frequent methodological design was consecutive retrospective cohort of patients with 6 studies [3, 10, 17, 20, 22, 23]. Two studies were case-control studies [5, 23] and three studies were nested cohorts within registries (European registry IDES [21], Danish registry [7], Swedish registry [24]).

### 3.2. Quality of Available Evidence

To assess quality of evidence and establish the feasibility of meta-analysis, we focused on four main criteria: sample size, risk of bias, definition of interventions, and definition of outcomes.

The included studies enrolled a total of 64,988 patients. Sample sizes were quite variable across the studies and ranged from 48 [17] to 42.238 [24] patients, with less than 1000 patients in most of the studies. Sample size is a critical issue for an accurate evaluation of differences between staged and simultaneous surgeries in the outcomes of interest. Considering the low incidence in most of the outcomes (e.g., 90-day mortality: 0.7% [25], pulmonary embolism: 0.9% [26], deep infection: 0.2% [26]) a large number of patients are required to have power to detect differences. We did not calculate the optimal sample size for each one of the outcomes of interest, but the study of Garland et al. [24] estimated that 1,346 patients with simultaneous bilateral THA and 13,460 controls would be required in order to detect a twofold increase in mortality, assuming that 90-day mortality is 0.7%; comparable sample sizes would be required for other outcomes with similar incidences (e.g., DVT, pulmonary embolism, deep infection). With these numbers, only two studies [23, 24] exceeded the optimal sample size to have power to detect differences in the main outcomes.

#### 3.2.1. Selection Bias

**Figure 2:**
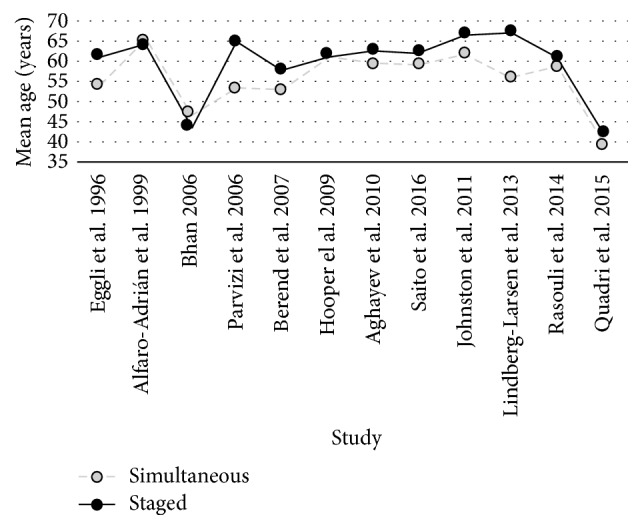
Summary of mean age in the different groups in selected articles.

Only one study [27] used randomization to allocate patients to staged or simultaneous surgery; in the rest of the studies the allocation was done by clinical decision between surgeons and patients; this choice was directly related to the outcomes of interest (i.e., younger patients less prone to complications are more likely to be allocated to simultaneous surgeries), thus revealing a clear selection bias.  Figure 2 shows how the simultaneous surgery group was younger in most of the studies, with a statistically significant difference in mean age between groups in six studies [7, 11, 16, 18, 20, 22].

Comorbidities were reported in 9 studies [3, 11, 17, 19–24]; several systems were used to classify them including the ASA classification system, the Charnley system, the Elixhauser system, and the number of comorbidities. In four studies [17, 20, 28, 29], a greater burden of comorbidity was found in the staged surgery group, whereas in three other studies [3, 19, 22] the greater comorbidity was found in the simultaneous group. In one study [21] patients with comorbidities (Charnley type C) were excluded. Although there was no tendency to allocate the sickest patients to a single group (staged or simultaneous), there was no control of this confounder variable across most of the studies.

#### 3.2.2. Confounders

Potential confounders of effects between the surgical strategy (staged or simultaneous) and outcomes of interest such as age, comorbidities, and cointerventions were poorly controlled in most of the studies.

As earlier noted, age and comorbidities were different between groups, and these variables were controlled with multivariate regression models only in two studies [23, 24].

*Intervention*. The type of arthroplasty and surgical approach were variable across the studies. The operative and postoperative management protocol were reported inconsistently, and when reported, they were widely variable.

The simultaneous surgery term was used for bilateral one-stage surgery during one anesthesia event. The “staged” group term was used for sequential surgeries in more than one anesthesia event; this definition yielded a very heterogeneous group since the time elapsed between the first and the second hip was quite variable; one study [23] with both surgeries performed in a single hospitalization (1 to 35 days of interval) was found, whereas the rest of the studies were carried out using different hospitalizations between surgeries with an interval that ranged from 2 weeks to 5 years. Theoretically, the risk of complications could change over the time after the first surgery due to physiological stress; this could pose differential subgroups basal risks for the outcomes of interests in a supposed “same” population (e.g., different risks if time elapsed between surgeries is 1 day versus 6 weeks). Therefore, it is desirable to have stratified subgroup analysis according to the time elapsed between surgeries; in this revision only four studies [7, 18, 21, 24] performed stratified analyses by subgroups according to time between the first and second hip for the mortality outcome.

The follow-up period of the studies was variable; however, all have a follow-up greater than 6 months, a time lapse that is sufficient to detect the outcomes of interest for this review.

- Definition of outcomes
  - Mortality: five studies [3, 10, 11, 23, 24] reported data on mortality. Mortality was measured at different times in each study: in-hospital mortality in the Rasouli et al. study [23], 30-day mortality in the Hooper et al. study [10], 90-day mortality in the Garland et al. study [24], and mortality at 6 months in the Parvizi et al. study [28]. Only the Garland et al. study [24] presented a Cox survival model adjusting mortality to variables that may confound the effect of timing of surgeries on mortality.
  - Thromboembolic disease: six studies [3, 5, 10, 11, 18, 19] reported discriminated data for DVT and/or pulmonary embolism. None of the studies approaches the optimal sample size to have power to detect differences in this outcome. The measurement of these outcomes was variable (Doppler, venography, ventilation/perfusion scintigraphy, patient report) and, in most cases, was not described. The use of thromboprophylaxis was widely variable using aspirin [19], warfarin [3, 28], or enoxaparin [17] for variable time; moreover, in some cases pharmacological thromboprophylaxis was not used [5]. Thromboembolic disease has known risk factors and treatment can influence its incidence; therefore it is desirable to adjust for these variables to detect without confusion the effect of the stage surgeries on this complication.
  - Cardiovascular complications: only three studies presented discriminated data for cardiovascular complications in general, without differentiating any specific cardiovascular complication. The definition of these outcomes and their measurement was not specified in the studies.
  - Other major complications: most of the studies presented combined outcomes, dividing the complications into systemic and local and grouping by systems (gastrointestinal, neurological, urinary, pulmonary).
  - Bleeding: total bleeding was the outcome measured in all studies; however, the measurement was heterogeneous across studies, using different definitions such as weight of compresses, drainage of suction systems, hemoglobin/hematocrit decline, or drainage within the first 24 hours. Some studies for two-stage surgery used an average bleeding of the two surgeries, while others did a cumulative analysis.
  - Transfusion: it was measured homogeneously in the studies as the number of units transfused; however, the decision of transfusion was variable and at clinical discretion in all the studies. Cointerventions such as cell-saver and autotransfusion were used in some studies, thus increasing the heterogeneity.

In summary, due to high risk of bias in most of the studies, heterogeneity in the time elapsed between surgeries in the staged group, heterogeneity in cointerventions, and variability in the definition and measurement of outcomes, we considered it inappropriate to perform a meta-analysis. Therefore, only the results of the systematic review are reported.

### 3.3. Results of Systematic Review

#### 3.3.1. Summary of Findings of Best Available Evidence

According to the risk of bias and potential confounders we decided to present the results of the literature with the lowest risk of biased effects (sample sizes with large number of patients, control of confounders in regression models). Summary of the findings of the systematic review is presented in Table 3.

*(i) In-Hospital Mortality*. Rasouli et al. [23] evaluated in-hospital mortality, using a national database with 16330 patients in total, 14798 patients in the simultaneous surgery group (mean age 58.43 ± 13.77) and 1532 patients in staged surgery (mean age 60.29 ± 12.1) performed in the same hospitalization between 1 and 35 days after the first surgery, finding a* mortality rate of 0.1% for the simultaneous surgery group and 0.3% for the staged surgery group, difference that was not statistically significant.*

*(ii) Ninety-Day Mortality*. Garland et al. study [24] compared the 90-day mortality of patients who received simultaneous bilateral total hip replacement with patients who underwent staged surgery stratified into three groups (less than 6 months, 6 months to 1 year, and over 1 year) in a cohort of 42,238 patients nested in the Swedish registry of arthroplasties. The group of patients who were treated with simultaneous bilateral were younger and healthier.

To evaluate mortality risk, Kaplan Meir survival curves were performed and hazard ratios (HR) were calculated using a Cox model adjusted for sex, age, diagnosis, and type of fixation of the prosthesis, finding that the risk of death within* 90 days after the second procedure was not different among the four groups investigated.*

However, in simultaneous procedures patients older than 75 years old (OR 3.8 CI 95% 2.6–5.6), with rheumatoid arthritis (OR 2.3 CI 95% 1.1–4.7) or with ASA 3 or more (OR 8.2 IC 95% 1.8–37.3) adjusting for the remaining variables had a significantly higher risk of mortality compared with the rest of the cohort. It therefore suggests that special attention should be given to this population of patients when dealing with bilateral hip osteoarthrosis.

*(iii) Combined Systemic and Local Complications*. The aforementioned study by Rasouli et al. [23] compared the risk of presenting systemic complications, as a composite outcome, among the groups of simultaneous surgery (mean age 58.43 ± 13.77 years) and staged surgery (mean age 60.29 ± 12.1) performed in the same hospitalization between 1 and 35 days, using a logistic regression model controlling age, gender, comorbidity index, type and size of hospital, type of insurance, geographic region, year, and comorbidities (coronary disease, coagulopathy, peripheral vascular disease, renal disease, obesity, diabetes mellitus, lung disease, congestive heart failure, pulmonary vascular disease);* no statistically significant difference was found between the two groups (OR 0.84 95% CI 0.63*–*1.1).*

The combined systemic complications were central nervous system, cardiac, respiratory, gastrointestinal, genitourinary complications; postoperative shock; deep venous thrombosis; pulmonary embolism; acute anemia.

The risk of presenting combined local complications was assessed in the same population and adjusted for the same variables in a regression model,* finding that bilateral staged surgery in the same hospitalization might be associated with an increased risk of local complications compared to simultaneous surgery (OR 1.75, 95% CI 1.00*–*3.07).*

The local complications combined were complications related to the devices, hematoma/seroma, accidental puncture/laceration, surgical wound dehiscence, surgical wound infection.

*(iv) Bleeding and Transfusion*. The best available evidence for this outcome with a very low risk of bias is given by the only randomized clinical trial from this review. Bhan et al. [27] randomized 168 patients, 83 had a simultaneous and 85 a two-stage procedure (at least 3 months after the first procedure), with a mean age of 46.59 (SD 14.98) and 43.38 (SD 14.35), respectively. Sample size was estimated to show a difference in postoperative hemoglobin level of 0.5 g/dl with an alpha level of 0.05 and a power of 80%, for other outcomes this study is underpowered. For patients in the two-stage group, the total blood loss was calculated as the sum of the values for each operation.* The mean total blood loss was significantly higher in the two-stage group compared with that in the simultaneous group* (1997.06 ml (SD 490.78) and 1473.86 ml (SD 517.14), resp.) (*p* < 0.001);* however, the mean number of blood units transfused was significantly lower in the two-stage group*, considering that the total blood loss for the simultaneous group is at one point posing a higher physiological stress. Likewise, the* postoperative hematocrit at eight hours was significantly lower in the simultaneous group, 0.287 (SD 0.051) as compared with 0.321 (SD 0.045) in the two-stage group *(*p* = 0.024). Care should be taken when extrapolating the results from this study to older patients, because this study population is younger than the usual patient with hip osteoarthrosis. Although the mean total blood loss and postoperative hematocrit could be similar in older patients, the impact of these in physiological responses and cardiovascular outcomes could be different.

Recommendations based on available evidence

- Although most of available evidence is affected by selection bias, there is no difference in the risk of complications in young and healthy patients. Therefore, available evidence supports simultaneous bilateral surgery if the following characteristics are met:
  - patients not older than 75 years [24],
  - patients with low anesthetic risk ASA 1-2,
  - patients without cardiovascular comorbidities or rheumatoid arthritis.
- In patients who require bilateral hip surgery and are not good candidates for simultaneous surgery by the criteria previously described, staged surgery during the same hospitalization must be avoided due to an increased risk of complications.

## 4. Discussion

In this study, we addressed two questions: Is it safer to perform a simultaneous or staged bilateral hip arthroplasty? And if staged, how long should surgeons wait to operate the second hip without increasing risks? After reviewing the literature, two conclusions were obtained.

First, available evidence has a low quality and it is very heterogeneous. To date, clinical studies have focused on finding differences between simultaneous or staged surgery, in most cases affected by selection bias, differences in confounders between populations, the lack of standardization of outcomes, and ignoring the fact that it is inappropriate to group staged surgeries without subgroups analysis considering the time lapse between procedures. Therefore, the vast majority of published studies are not homogeneous with regard to the age and comorbidities between the groups [7, 10, 20, 22, 30] or the time elapsed between procedures in the staged surgery groups [7, 10, 19, 21, 23, 31, 32], reflecting a clear selection bias as younger and healthier patients are more frequently allocated to simultaneous surgery, which threatens internal validity of studies, and heterogeneous basal risks, which threatens the external validity of studies. Our group had stated these limitations of available evidence in a short communication before [14]. Although evidence has shown such serious weaknesses, two meta-analyses have attempted to perform a pooled analysis of evidence concluding that there is no difference between one- or two-stage surgery, without considering the clinical heterogeneity of the studies, the high risk of bias of the studies, and specially subgroups with differential risks [13, 33]. Considering this situation, a meta-analysis may not be appropriate since studies are not comparable, and, therefore, it is more accurate to report the findings as a systematic review; including those studies due to their design and quality could produce a valid conclusion. To our knowledge, this is the first study which states that a meta-analysis is not appropriate and reports the analysis of evidence as a systematic review, thus accepting the limitations of this design.

The second conclusion is that, in the clinical scenario, simultaneous bilateral surgery is a safe procedure when performed in selected patients. Individuals younger than 75 years old, without risk factors, such as cardiovascular disease or rheumatoid arthritis, and with low comorbidities (ASA 1-2) seem to have a low risk of complications when undergoing single stage procedures. This conclusion is supported mainly on the studies by Garland et al. [24] and Rasouli et al. [23] who reported no significant differences in complications between one- and two-stage surgeries; however, conclusions from these authors should be applied carefully because population in the simultaneous group was younger and healthier. On the other hand, for patients who are older than 75 or those with important comorbidities, there is no evidence clearly supporting the safety of the simultaneous surgery and there are reasons to expect increased risks with a demonstrated higher mortality in the bilateral surgery setting [24]. However, although a two-stage strategy seems to be safer than a one-stage approach, the risk of complications increases when procedures are performed in different stages during the same in-hospital stay [31]. The available evidence does not answer the question about what the best time between surgeries is when the staged strategy is selected; according to the existing evidence simultaneous bilateral procedure is suitable for young, healthy patients. Although, this data does not allow us to determine the time to operate the second hip, it suggests that the first week has a higher mortality rate.

## Figures and Tables

**Table 1:** Quality assessment for observational studies.

	Domains							
	Bias due to confounding	Bias in selection of participants in the study	Bias in classification of interventions	Bias due to deviations from intended intervention	Bias due to missing data	Bias in measurement of outcomes	Bias in selection of the reported result	Overall risk of bias
Eggli et al. 1996	Critical	Critical	Serious	Moderate	Low	Moderate	Moderate	*Critical*
Alfaro-Adrián et al. 1999	Critical	Critical	Serious	Moderate	Low	Moderate	Moderate	*Critical*
Parvizi et al. 2006	Critical	Critical	Serious	Moderate	Low	Moderate	Moderate	*Critical*
Berend et al. 2007	Critical	Critical	Serious	Moderate	Low	Moderate	Moderate	*Critical*
Hooper et al. 2009	Critical	Critical	Moderate	Moderate	Low	Serious	Moderate	*Critical*
Aghayev et al. 2010	Critical	Critical	Moderate	Moderate	Low	Moderate	Moderate	*Critical*
Saito et al. 2016	Critical	Critical	Moderate	Moderate	Low	Moderate	Moderate	*Critical*
Johnston et al. 2011	Critical	Critical	Moderate	Moderate	Low	Moderate	Moderate	*Critical*
Lindberg-Larsen et al. 2013	Serious	Critical	Moderate	Moderate	Low	Moderate	Moderate	*Serious*
Rasouli et al. 2014	Moderate	Moderate	Moderate	Moderate	Low	Moderate	Moderate	*Moderate*
Garland et al. 2015	Moderate	Moderate	Moderate	Moderate	Low	Low	Low	*Moderate*
Quadri et al. 2015	Critical	Critical	Serious	Moderate	Low	Serious	Moderate	*Critical*

**Table 2:** Quality assessment for experimental study.

	Domains					
Study	Random sequence generation (selection bias)	Blinding of participants and researchers (performance bias)	Blinding of outcome assessment (detection bias)	Incomplete outcome data (attrition bias)	Selective reporting (reporting bias)	Overall risk of bias
Bhan 2006	Low	Low	High	High	Low	*High*

**Table 3:** Summary of selected articles including evidence quality assessment and results of the studies.

Study	Type of Study	Sample Size	Surgery definition	Mean age (years)		Quality of evidence	Findings (safety outcomes)
				Simultaneous	Staged		
Eggli et al. 1996	Retrospective cohort	255	(i) Simultaneous (ii) Staged less than 6 weeks (iii) Stage 6 weeks and 6 months	54	61,3	Very low (i) Low power (ii) Selection bias	No difference between 3 groups

Alfaro- Adrián et al. 1999	Retrospective cohort	202	(i) Simultaneous (ii) Staged 2 weeks and 2 months	65	63,9	Very low (i) Low power	No difference between groups

Parvizi et al. 2006	Retrospective cohort	196	(i) Simultaneous (ii) Staged 25 days and 10 months	53	65	Very low (i) Low power (ii) Selection bias	No difference between groups

Bhan 2006	Randomized clinical trial	168	(i) Simultaneous (ii) Staged 3 and 7 months	46,5	43,8	Moderate (i) Low power	More bleeding in staged surgery, more transfusion for simultaneous surgery; no difference in complications

Berend et al. 2007	Retrospective cohort	277	(i) Simultaneous (ii) Staged 2 weeks and 2 years	52,7	57,3	Very low (i) Poor definition for staged surgery	More complications in simultaneous surgery

Hooper et al. 2009	Register, nested cohort	2092	(i) Simultaneous (ii) Staged without report of interval between surgeries	61	61	Very low (i) Selection bias	No difference between groups

Aghayev et al. 2010	Register, nested cohort	1819	(i) Simultaneous (ii) Staged less than 6 months (iii) Staged 6 months and 5 years	59	62	Very low (i) Poor outcome definition	No difference between groups

Saito et al. 2016	Retrospective cohort	89	(i) Simultaneous (ii) Staged in the same hospitalization	59	61,9	Very low (i) Low power	No difference between groups

Johnston et al. 2011	Retrospective cohort	594	(i) Simultaneous (ii) Stage without report of interval between surgeries	61,5	66,5	Very low (i) Selection bias	More adverse events in simultaneous surgery

Lindberg-Larsen et al. 2013	Register, nested cohort	680	(i) Simultaneous (ii) Staged less than 6 months (iii) Staged 6 months and 18 months	55,7	66,8	Low (i) Selection bias	No difference between groups

Rasouli et al. 2014	Retrospective cohort	16330	(i) Simultaneous (ii) Staged in the same hospitalization (1 to 35 days)	58,4	60,3	Moderate (i) Selection process not clear	More complications in staged surgery during the same in-hospital stay

Garland et al. 2015	Register, nested cohort	42238	(i) Simultaneous (ii) Staged less than 6 months (iii) Staged 6 months and 1 year (iv) Staged more than 1 year	<50 years = 18,3%	<50 years = 3%, 5,3%, 7,8%	Moderate (i) Selection bias	Simultaneous surgery is safe in young and healthy patients

Quadri et al. 2015	Retrospective cohort	48	(i) Simultaneous (ii) Staged without report of interval between surgeries	39	42	VERY LOW (i) Low Power (ii) Selection Bias	No difference between groups

## Appendix

### A. MEDLINE Search Strategy

((”Arthroplasty, Replacement, Hip”[Mesh] AND ((”bilateral” OR ”staged”) OR ”two-stage”) OR ”one-stage” OR ”simultaneous”)) NOT ”infected” NOT ”infection.”

### B. Embase Search Strategy

'total hip prosthesis':ab,ti AND bilateral:ab,ti OR ('total hip prosthesis':ab,ti AND 'simultaneous':ab,ti) OR ('total hip prosthesis':ab,ti AND 'staged':ab,ti) OR 'bilateral hip' AND [embase]/lim NOT [medline]/lim AND ('human'/exp OR 'human').

### C. CENTRAL Search Strategy

MeSH descriptor: [Arthroplasty, Replacement, Hip] explode all trees AND ”bilateral”:ti,ab,kw OR ”stage”:ti,ab,kw OR ”simultaneous”:ti,ab,kw.
